# Local Anesthesia Versus Local Anesthesia and Conscious Sedation for Inguinal Hernioplasty: Protocol of a Randomized Controlled Trial

**DOI:** 10.2196/resprot.6754

**Published:** 2017-02-07

**Authors:** Pierre-Anthony Leake, Patrick J Toppin, Marvin Reid, Joseph M Plummer, Patrick O Roberts, Hyacinth Harding-Goldson, Michael E McFarlane

**Affiliations:** ^1^ Department of Surgery, Radiology, Anaesthesia and Intensive Care Faculty of Medical Sciences University of the West Indies Kingston Jamaica; ^2^ Tropical Medical Research Institute University of the West Indies Kingston Jamaica

**Keywords:** inguinal hernioplasty, local anesthesia, conscious sedation, patient satisfaction, randomized controlled trial

## Abstract

**Background:**

Conscious sedation is regularly used in ambulatory surgery to improve patient outcomes, in particular patient satisfaction. Reports suggest that the addition of conscious sedation to local anesthesia for inguinal hernioplasty is safe and effective in improving patient satisfaction. No previous randomized controlled trial has assessed the benefit of conscious sedation in this regard.

**Objective:**

To determine whether the addition of conscious sedation to local anesthesia improves patient satisfaction with inguinal hernioplasty.

**Methods:**

This trial is designed as a single-center, randomized, placebo-controlled, blinded trial of 148 patients. Adult patients diagnosed with a reducible, unilateral inguinal hernia eligible for hernioplasty using local anesthesia will be recruited. The intervention will be the use of intravenous midazolam for conscious sedation. Normal saline will be used as placebo in the control group. The primary outcome will be patient satisfaction, measured using the validated Iowa Satisfaction with Anesthesia Scale. Secondary outcomes will include intra- and postoperative pain, operative time, volumes of sedative agent and local anesthetic used, time to discharge, early and late complications, and postoperative functional status.

**Results:**

To date, 171 patients have been recruited. Surgery has been performed on 149 patients, meeting the sample size requirements. Follow-up assessments are still ongoing. Trial completion is expected in August 2017.

**Conclusions:**

This randomized controlled trial is the first to assess the effectiveness of conscious sedation in improving patient satisfaction with inguinal hernioplasty using local anesthesia. If the results demonstrate improved patient satisfaction with conscious sedation, this would support routine incorporation of conscious sedation in local inguinal hernioplasty and potentially influence national and international hernia surgery guidelines.

**Trial registration:**

Clinicaltrials.gov NCT02444260; https://clinicaltrials.gov/ct2/show/NCT02444260 (Archived by WebCite at http://www.webcitation.org/6no8Dprp4)

## Introduction

Inguinal hernia repair is one of the most commonly performed surgical procedures in the world. It is estimated that 20 million inguinal hernia repairs are performed globally every year [1]. Inguinal hernia repair prevents the development of complications of inguinal hernias such as incarceration, obstruction, and strangulation with their ensuing morbidity and mortality. Mesh repair (ie, hernioplasty) is superior to suture repair and is now standard for inguinal hernia repair [2].

There are a variety of anesthetic options available for this procedure. These include infiltration of the inguinal region using local anesthesia (LA), the use of regional anesthesia such as a subarachnoid block, or general anesthesia. For suitable inguinal hernias, the use of LA has been a simple and very popular option [3]. Several high-quality studies have suggested numerous advantages of LA over both regional and general anesthesia techniques. These include shorter operative time [4], reduced nausea [4,5], less urinary retention [4,6,7], increased day-case rates [6,7], less postoperative analgesia [6,7], faster return to normal activity [5], reduced costs [8], and greater patient satisfaction [4,9]. Patient tolerance and satisfaction for hernioplasty performed using LA may be as high as 82%-98% [4].

One adjunct that is utilized to improve patient experience with ambulatory surgical procedures is the addition of conscious sedation. Conscious sedation refers to the use of sedative or dissociative agents and analgesics to induce a state that enhances patient tolerance of unpleasant procedures while maintaining cardiorespiratory function. Use of sedation in addition to LA for surgical procedures allays patient anxiety, reduces autonomic arousal, and often provides amnesia for the procedure. However, evidence linking conscious sedation to improved patient satisfaction and its effect on other patient outcomes has been lacking.

Potential issues with the use of conscious sedation include delayed awakening and impairment of psychomotor function that may prolong postoperative recovery and reduce the rate of same-day discharge [10,11]. These issues initially limited the use of sedation for inguinal hernioplasty using LA. The use of midazolam, with its high therapeutic index and relatively short duration of action, has allayed these concerns [12]. This high therapeutic index has facilitated the use of midazolam for sedation by nonanesthetists with the presence of appropriate monitoring. Inguinal hernioplasty using LA with conscious sedation (LACS) has demonstrated acceptable patient outcomes with no mortality and acceptable morbidity [13-16]. A study from the University of the West Indies prospectively evaluated 90 patients undergoing inguinal hernioplasty using LA combined with midazolam. Operative morbidity was low, with all patients discharged ambulant and same day. No adverse reactions to sedation were demonstrated [17].

Inguinal hernioplasty using either LA or LACS has demonstrated good patient outcomes, patient satisfaction, and low morbidity. No study has directly compared LA to LACS in patients undergoing inguinal hernioplasty in order to establish superiority of either method.

It was hypothesized that the addition of conscious sedation to local anesthesia would result in improved patient satisfaction with no change in the rate of same-day discharge or perioperative complications when compared to the use of LA alone.

## Methods

### Trial Design

The trial will be a single-center, randomized, blinded, placebo-controlled trial of 148 participants with an allocation ratio of 1:1. Patients, surgeons, outcome assessors, and data analysts will be unaware of the treatment assignments.

### Primary and Secondary Endpoints

The primary endpoint of this trial is patient satisfaction with the method used: anesthesia alone or anesthesia plus sedation. Patient satisfaction will be assessed using the Iowa Satisfaction with Anesthesia Scale (ISAS) [18]. ISAS is a validated scale consisting of 11 parameters measured on a 6-point Likert scale. ISAS has been used to assess patient satisfaction with anesthesia across various surgical specialties, including ophthalmology and orthopedic surgery, and for various types of anesthesia. It will be administered at the time of discharge from hospital following the surgical procedure and at the 2-week follow-up visit.

The secondary endpoints include measurement of pain during and following the procedure, the quantity of anesthetic and/or sedation required, the frequency of complications, the frequency of functional incapacity [19], and the time to discharge [20] from hospital. A complete list of these secondary endpoints is provided in Table 1.

**Table 1:** Complete list of secondary endpoints.

Study endpoint	Definition
Intraoperative pain	Patient’s perception of pain felt during the procedure as measured by a visual analog scale
Postoperative pain	Patient’s perception of pain being experienced at the time of discharge as measured by a visual analog scale
Operative time	Time from incision to wound closure
Volume of local anesthetic	Total volume of local anesthetic administered during the procedure
Volume of conscious sedation	Total volume of conscious sedative agent administered during the procedure
Time to discharge	Time from transfer to the recovery room to scoring at least 9 out of 10 on the Modified Post-Anesthetic Discharge Scoring System [20]
Frequency of early postoperative complications	Any postoperative complication occurring within 30 days of the surgical procedure, including wound hematoma, scrotal hematoma, surgical site infection, seroma, and wound dehiscence
Frequency of late postoperative complications	Any postoperative complication occurring between 30 days and 1 year following the surgical procedure, including chronic pain, hydrocele, and recurrence
Frequency of functional incapacity	Impairment in functional abilities following the surgical procedure as measured by a 13-parameter, 6-point Likert scale by McCarthy et al [19]

### Inclusion and Exclusion Criteria

The trial (ClinicalTrials.gov identifier: NCT02444260; protocol number: ECP 342, 12/13) was approved by the Ethics Committee of the Faculty of Medical Sciences, University of the West Indies at Mona. All patients will be informed about the purpose of the trial, the two study arms, and the potential benefits and risks. Eligible patients will be 18-75 years of age and diagnosed with a reducible inguinal hernia. Patients will be selected solely based on suitability for inguinal hernioplasty with local anesthesia and not because of easy availability, diminished autonomy, or social bias. Exclusion criteria are listed in Textbox 1.

**Exclusion criteria for the study.**

Exclusion criteria:

- Age less than 18 years or greater than 75 years
- Known renal, hepatic, respiratory, cardiovascular, neurologic, or psychiatric disease
- Body mass index >30 kg/m^2^
- Bilateral, recurrent, inguinoscrotal, or incarcerated/irreducible hernia
- Allergy to local anesthetic or sedative agents
- Pregnancy, lactating, or breastfeeding patients
- Chronic pain syndromes, such as sickle cell disease
- Anxiety disorder
- Regular opioid analgesia or sedative use
- Serious medical condition likely to impede successful completion of the study
- Current participation in other therapeutic clinical trials

### Determination of Sample Size

Assuming a variance of 1.3, as established by Dexter et al [18], a sample size of 74 participants per subject group (ie, a total of 148 participants) would provide 80% power to detect a difference in ISAS score of 0.6 at 5% significance between the two subject groups—LA versus LACS. These values were derived from data by Candiotti [21] in a randomized controlled trial showing a 0.6 ISAS score difference between two intervention arms. To mitigate the effects of dropouts, we proposed to recruit 90 subjects per group for a total of 180.

### Study Procedures

**Figure 1:**
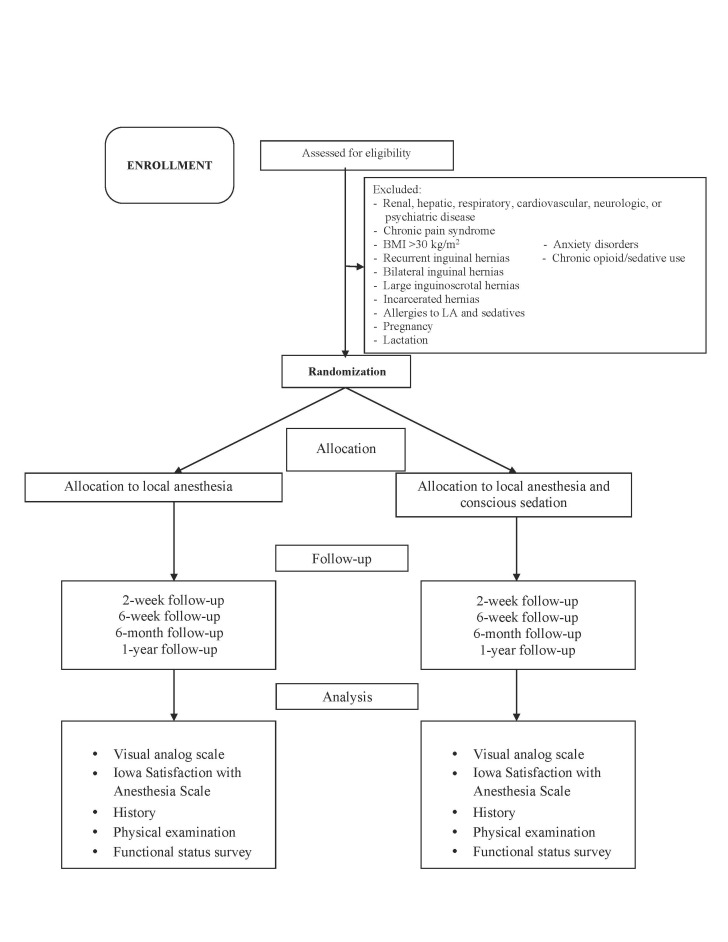
Flowchart summarizing study procedures and participant flow through each stage. BMI: body mass index; LA: local anesthesia.

The study procedures and participant flow through each stage are summarized in the flowchart in Figure 1.

#### Screening and Recruitment

Subjects will be recruited as they are evaluated in the surgical outpatient department (SOPD) at the University Hospital of the West Indies (UHWI). Clinical staff will review new patients referred to the SOPD for management of inguinal hernias and those previously seen but awaiting surgical management. Following initial clinical assessment for study suitability by the clinical staff, the research nurse coordinating the study will approach potential study patients and assess them for participation. These potential subjects will complete an eligibility-screening questionnaire to document fulfillment of the entry criteria. Eligible patients will have the study explained to them and provided with full study information prior to consent being obtained. Patients will be given ample opportunity to review the information provided and have any questions answered prior to making the decision to consent. Once the informed consent document is signed, the subject will be considered *registered* in the study. Patients that are not eligible for the study and eligible patients that decline to participate in the study will continue the usual assessment and therapy as dictated by the practice guidelines of UHWI.

#### General Scheduling

Once consent is given, a study number will be assigned and this will be used to identify the subject throughout the study. Once registered, scheduling of study evaluations will proceed. Patients will be sent to the minor operating theater of the UHWI where a date for surgery will be issued and surgical preparation instructions given. A total of six visits are scheduled for the trial (see Table 2). Follow-up visits will be carried out at 2 weeks, 6 weeks, 6 months, and 1 year where patients will be examined and issued questionnaires evaluating patient satisfaction, pain evaluation, and functional status [19].

**Table 2:** Schedule of visits during the course of the trial.

Study step	Visit					
	1 (SOPD^a^)	2 (Operation)	3 (2 weeks postop^b^)	4 (6 weeks postop)	5 (6 months postop)	6 (1 year postop)
Demographics and baseline clinical data collected	X	X				
Eligibility criteria determined	X					
Randomization performed		X				
History and clinical examination performed			X	X	X	X
Visual analog scale administered		X	X			
Iowa Satisfaction with Anesthesia Scale administered		X	X			
Functional status determined			X	X	X	X

^a^SOPD: surgical outpatient department.

^b^postop: postoperation.

#### Randomization

The study will be blinded. Randomization will be done using the *ralloc* command in Stata (StataCorp LLC) version 14. In order to reduce the risk of breaking the blind because of small block sizes or alternately to prevent unequal sample sizes if the trial is stopped prematurely, treatment allocation will be done according to a 1:1 ratio and block sizes will be allocated in proportion to elements of Pascal’s triangle. The ensuing allocation table will be kept in a secure file on the study statistician’s computer and will be made available to the anesthetist on a per-enrolled-patient basis when required in a sealed envelope immediately prior to the procedure.

#### Preoperative Assessment and Preparation

On the day of their procedure, all patients will be seen in the holding area. A presurgery checklist will be completed to ensure that the patient has no immediate contraindications to surgery. All parameters must be within specified ranges in order to proceed. If there are abnormalities that preclude safe conduct of the procedure, the procedure will be postponed and the patient returned to the SOPD. Reevaluation for participation in the study will be possible once the identified abnormality is corrected. The patient will have to reenter the study at the eligibility-screening phase, though the study identifier will remain the same.

#### Standardized Treatment

Perioperative and intraoperative care will be standardized, except for the administration of conscious sedation, which will be randomized by the attending anesthetist according to the allocated study group. Prophylactic antibiotics will not be administered.

Local anesthesia will be administered by the surgeon and will be used in all cases. A mixture of 1% lignocaine and 0.25% bupivacaine to a maximum of 4.5 mg/kg and 2 mg/kg, respectively, will be infiltrated. An initial dose will be administered prior to skin incision with subsequent doses administered during the procedure. Protocols for rescue intraoperative analgesia have been established in the event that the maximum amount of local anesthetic has been infiltrated without effective pain control.

The operative technique will be a standardized Lichtenstein repair using polypropylene mesh. A single consultant surgeon will be supervising surgical residents. During the procedure, the ilioinguinal nerve will be spared.

#### Trial Interventions

Following placement on the operating table and the attachment of monitoring devices, the anesthetist will open the randomization envelope. Only she or he will know the allocated study intervention.

In the LACS group, the sedative agent being used will be midazolam. This will be administered at an initial dose of 2 mg, with incremental doses of 0.5-1 mg to a maximum of 10 mg. Additional doses of midazolam will be administered based on assessment of the patient’s level of sedation at 15-minute intervals using the Ramsay Sedation Scale [22] (see Table 3). A score of 2-3 on the Ramsay Sedation Scale will be maintained. In the LA group, the anesthetist will administer placebo (ie, intravenous normal saline) to the patient in a similar manner to the midazolam in order to maintain blinding.

**Table 3:** The Ramsay Sedation Scale scores and their definitions.

Score	Definition
1	Anxious and agitated, restless, or both
2	Cooperative, oriented, and calm
3	Responsive to commands only
4	Exhibiting brisk response to light glabellar tap or loud auditory stimulus
5	Exhibiting a sluggish response to light glabellar tap or loud auditory stimulus
6	Unresponsive

#### Blinding

The patient and surgeon will be blinded to the randomization. We recognize that both the patient and surgeon may have an opinion as to the randomization based on known effects of sedative agents (ie, sleep). However, neither will be made aware directly of the allocation. The patient will be draped in such a manner that the surgeon will not be able to see the patient during the procedure. Conversation between patient and surgeon during the procedure will be discouraged. The allocation of the participant to the study group will be unmasked prior to the end of the trial only if a serious adverse event occurs.

### Outcome Assessment and Follow-Up

#### At Discharge

Patient demographic data and medical history, operative time, volume of local anesthetic, and volume of conscious sedation used (if applicable) will be recorded. Following inguinal hernioplasty, the subject will be transferred to the recovery room where she or he will be monitored by nursing staff. The nursing staff will be trained to determine the subject’s readiness for discharge based on specified discharge criteria [20]. The discharge criteria questionnaire will be administered 1 hour after admission to the recovery room and thereafter at 30-minute intervals until the patient has achieved the appropriate score for discharge. Once the subject has fulfilled the criteria for discharge, the time of discharge will be recorded on the subject’s research operative chart. A questionnaire assessing surgical experience and satisfaction will be administered at the time of discharge. It will include a previously validated visual analog pain scale [23] and the Iowa Satisfaction with Anesthesia Scale [18].

Prior to discharge, all patients will be given a supply of oral analgesics, which will include acetaminophen and diclofenac. Instructions on the dosages and administration schedule for these drugs will be included with the medications. Patients will be given a telephone number to contact if they have any questions about postoperative analgesia administration or instructions.

#### Follow-Up Visits

Subjects will return for routine interval follow-up visits 2 weeks, 6 weeks, 6 months, and 1 year following the surgical procedure. The 1-year visit will be considered the study exit visit. At the time of discharge following surgery, the study coordinator will calculate the follow-up dates. These will be indicated in the subject’s research chart as well as the hospital chart. A special sticker will be placed on the hospital chart to indicate that the subject is involved in this study and should be seen by the study coordinator.

Table 2 shows the mode of assessment and the outcome measures evaluated at each follow-up visit.

### Assessment of Safety

From the time of consent until completion of the study at 1-year follow-up or premature withdrawal, all serious adverse events will be reported to the Ethics Committee and the Data Safety Monitoring Board within 24 hours of the principal investigator learning of its occurrence. All adverse events occurring from the time of consent until 30 days following completion of the last follow-up visit will be recorded; those at grade 2 or higher using the Common Terminology Criteria for Adverse Events version 4.0 [24] will be reported to the Ethics Committee and the Data Safety Monitoring Board. Information to be submitted will include the study identifier, the nature and grade of the adverse event, and the outcome or nature of the event.

The Data Safety Monitoring Board will be responsible for assessing adverse outcome data collected during the course of the trial at 6-month intervals and communicate findings to the Ethics Committee. Unacceptable findings will be an indication for early termination of the study.

### Data Management and Monitoring

Subjects enrolled in this study will be assigned and tracked according to a unique study identifier assigned at the time of the subject’s registration. A log report will be kept to track each subject that is registered. This log will be kept as a hard copy in the regulatory binder and stored securely in a locked cabinet in the office of the principal investigator. All data collected in the trial, including on the day of the procedure and during follow-up visits, will be collected on case report forms. The completed case report forms will be reviewed by the principal investigator and data from the forms will be transferred to a database on a secure computer. The case report forms will be stored as hard copies in a locked cabinet. All data are managed and analyzed at the Tropical Metabolic Research Institute, University of the West Indies.

Monitoring is carried out in accordance with the Declaration of Helsinki, International Conference on Harmonization Guidelines for Good Clinical Practice (codification E6) and standard operating procedures of the Faculty of Medical Sciences, University of the West Indies.

### Statistical Analysis Plan

Demographic variables and quantitative data will be displayed in tables. Summary measures of continuous variables will be expressed as means with standard deviation, minimum, and maximum. Categorical variables will be expressed as counts. Differences in mean values by group will be tested with an independent *t* test while differences in proportions for categorical variables by group will be assessed with chi-square statistics. A *P* value of <.05 will be considered significant.

### Dissemination of Results

Data will be collated, analyzed, and submitted for publication as soon as possible following completion of the study. The results of this study will be submitted for publication whether or not a significant difference is found in the outcomes for the two groups.

## Results

This trial has been designed as a single-center, randomized controlled trial and was initiated at the Department of Surgery, University of the West Indies at Mona, in February 2014. To date, a total of 171 patients have been recruited. Surgery has been performed on 149 patients, meeting the sample size requirements. Follow-up assessments are still ongoing. Trial completion, with completed follow-up, is expected in August 2017.

## Discussion

We present a study protocol for a randomized controlled trial to assess the effect of adding conscious sedation (ie, midazolam) to local anesthesia (ie, lignocaine and bupivacaine) on patient satisfaction with inguinal hernioplasty. Patient satisfaction is an indirect indicator of the quality of health care. Its impact extends beyond the patient to also include the hospital staff, the institution, and potentially national policy making. It influences patient loyalty and retention, patient and staff morale, staff productivity, and overall profitability [25].

Inguinal hernioplasty using local anesthesia alone has long been a standard for ambulatory hernia surgery, with reports of reasonable patient satisfaction [4]. With increasing use of conscious sedation in ambulatory surgery [26-28], it is prudent to establish its utility for ambulatory hernia surgery.

## Abbreviations

BMI: body mass index
ISAS: Iowa Satisfaction with Anesthesia Scale
LA: local anesthesia
LACS: local anesthesia with conscious sedation
postop: postoperation
SOPD: surgical outpatient department
UHWI: University Hospital of the West Indies
